# Supplementary material for: Content-rich biological network constructed by mining PubMed abstracts
Source: BMC Bioinformatics. 2004 Oct 8;5:147. doi: 10.1186/1471-2105-5-147 (PMC528731; doi:10.1186/1471-2105-5-147)
Supplement: Additional File 2 — The original results of the above study (non-essential files are deleted to keep the file size under the limit set by BMC bioinformatics). [file 1471-2105-5-147-S2.bz2 › chilibotAdditionalFile2/dip10/671ID11110785E11073/html/CCR3_CXCL10.html]

 


 **CCR3** and **CXCL10** 
  
Found 37 abstracts in PubMed, retrieved 10.  
 

 What does Google say? 
 PDF only 
| .edu only 

---

**Interactive relationship** (e.g. stimulation, inhibition, etc)

**Neutral relationship**- **CCR3**  functional responses are regulated by both CXCR3 and its ligands CXCL9,  **CXCL10**  and CXCL11.  Ref: 12884299 Eur J Immunol, 2003
- An unusual structural feature of IP 10  [ **CXCL10** ]  was identified that may provide the basis for the ability of IP 10  [ **CXCL10** ]  to bind both CXCR3 and  **CCR3** .  Ref: 12173928 Biochemistry, 2002
- Type 1 chemokines CXC chemokine ligand CXCL 9 and  **CXCL10**  monokine induced by IFN gamma and IFN gamma inducible protein of 10 kDa CXCR3 ligands, and type 2 chemokines CC chemokine ligand CCL 11 eotaxin  **CCR3**  ligand, CCL17 thymus and activation regulated chemokine CCR4 ligand and CCL22 monocyte derived chemokine CCR4 ligand were readily detectable in the plasma of most participants.  Ref: 12407016 Int Immunol, 2002

**Non-interactive relationship** (e.g. studied together, co-existance, homology, etc.)

- Both thymus and activation regulated chemokine TARC CCL17, a ligand for CC chemokine receptor 4 that is mainly expressed on Th2 type cells, and interferon induced protein of 10kDa IP 10  [ **CXCL10** ]   **CXCL10** , a ligand for CXC chemokine receptor 3  [ **CCR3** ]  that is mainly expressed on Th1 type cells, are produced by keratinocytes after the stimulation with the primary cytokines such as tumor necrotic factor alpha TNF alpha and or interferon gamma IFN gamma.  Ref: 12441140 Cytokine, 2002
- Consistent with these results, infected cells from WT mice preferentially migrated in response to IP 10  [ **CXCL10** ]  CXCR3 ligand, while those from GKO mice migrated in response to eotaxin CCL11  **CCR3**  ligand.  Ref: 12875978 Am J Pathol, 2003
